# Analysis of Myocardial Ischemia Parameters after Coronary Artery Bypass Grafting with Minimal Extracorporeal Circulation and a Novel Microplegia versus Off-Pump Coronary Artery Bypass Grafting

**DOI:** 10.1155/2020/5141503

**Published:** 2020-01-25

**Authors:** Luca Koechlin, Urs Zenklusen, Thomas Doebele, Bejtush Rrahmani, Brigitta Gahl, Thibault Schaeffer, Denis Berdajs, Friedrich S. Eckstein, Oliver Reuthebuch

**Affiliations:** Department of Cardiac Surgery, University Hospital Basel, Basel, Switzerland

## Abstract

**Background:**

To compare the performance of our institutionally refined microplegia protocol in conjunction with minimal extracorporeal circulation system (MiECC) with off-pump coronary artery bypass grafting (OPCAB).

**Methods:**

We conducted a single center study including patients undergoing isolated CABG surgery performed either off-pump or on-pump using our refined microplegia protocol in conjunction with MiECC. We used propensity modelling to calculate the inverse probability of treatment weights (IPTW). Primary endpoints were peak values of high*-*sensitivity cardiac troponin T (hs-cTnT) during hospitalization, and respective first values on the first postoperative day. Endpoint analysis was adjusted for intraoperative variables.

**Results:**

After IPTW, we could include 278 patients into our analyses, 153 of which had received OPCAB and 125 of which had received microplegia. Standardized differences indicated that treatment groups were comparable after IPTW. The multivariable quantile regression yielded a nonsignificant median increase of first hs-cTnT by 39 ng/L (95% CI -8 to 87 ng/L, *p* = 0.11), and of peak hs-cTnT by 35 ng/L (CI -13 to 84, *p* = 0.11), and of peak hs-cTnT by 35 ng/L (CI -13 to 84, *p* = 0.11), and of peak hs-cTnT by 35 ng/L (CI -13 to 84, *p* = 0.11), and of peak hs-cTnT by 35 ng/L (CI -13 to 84,

**Conclusion:**

The use of our institutionally refined microplegia in conjunction with MiECC was associated with similar results with regard to ischemic injury, expressed in hs-cTnT compared to OPCAB. MACCE was seen equally frequent. ICU discharge was earlier if microplegia was used.

## 1. Introduction

Despite a Ib recommendation for off-pump coronary artery bypass grafting (OPCAB) in patients with significant atherosclerotic aortic disease and a IIa recommendation for OPCAB for high-risk patients, OPCAB is not consistently applied [1, 2]. This is most probably due to the missing proof of long-term benefits of the OPCAB procedure since large randomized controlled trials failed to show a clear benefit for OPCAB procedures [3–7].

To reduce the potential negative side effects of the extracorporeal circulation systems (ECC), such as systemic inflammatory response syndrome or postoperative renal insufficiency, the minimal extracorporeal circulation system (MiECC) was introduced [8–10]. The use of the MiECC in coronary artery bypass grafting (CABG) was shown to be associated with excellent outcomes [11, 12]. Moreover, with regard to perioperative myocardial damage, the use of MiECC was shown to be comparable to OPCAB [11]. However, it is unknown to which extent the perioperative myocardial damage is dependent on the applied cardioplegic protocol. To further optimize MiECC in CABG surgery, we introduced the Myocardial Protection System (MPS®) in our clinic to deliver a novel microplegia solution [13]. This strategy, also referred to as Basel Microplegia Protocol (BMP), was shown to be beneficial regarding perioperative myocardial damage and length of stay on the intensive care unit (ICU) [14].

However, in comparison to OPCAB, the clinical role of the Basel Microplegia Protocol is undetermined. To better address this issue, we used observational data and performed a propensity modelling to calculate inverse probability of treatment weights (IPTW). These data were adjusted for possible confounding factors by indication.

## 2. Material and Methods

### 2.1. Ethical Approval

The local ethical committee (EKNZ BASEC Req-2018-00923) approved the study protocol, which is in accordance with the principles of the Declaration of Helsinki. The ethical committee has waived the need to obtain informed consent.

The trial was registered at ClinicalTrials.gov (ID NCT03609723). The authors designed the study, gathered and analyzed the data, vouched for the data and analysis, wrote the paper, and decided to publish.

### 2.2. Patients and Study Design (Figure 1)

The preferred revascularization strategies for isolated CABG surgery in our clinic were either OPCAB or MiECC-assisted surgery. In May 2017, we started to deliver our institutionally refined microplegia (Basel Microplegia Protocol) using the MPS® as an adjunct to the MiECC [13]. Since it performed excellently, this combination became a routine in isolated CABG with MiECC in our clinic [13, 14].

OPCAB is preferably used in patients with a high thromboembolic risk when manipulation of the aorta has to be minimized, in patients with severe renal insufficiency, and in patients with impaired myocardial function. Conventional ECC is only applied in emergency operations or concomitant non-CABG surgeries.

To investigate the effect of the two operation techniques (microplegia versus OPCAB) on the basis of our own observational data, we performed a propensity modelling to calculate inverse probability of treatment weights (IPTW) to adjust for possible confounding by indication.

Using a prospectively maintained institutional registry (Intellect 1.7, Dendrite Clinical Systems, Henley-on-Thames, UK), we identified all patients who underwent isolated CABG in our institution from February 2010 on. All clinical data were exported from this registry, where data are regularly controlled for completeness and accuracy [13, 14]. Intraoperative data were collected prospectively with a standardized protocol, and serological parameters were assessed according to the standard clinical algorithm in our hospital, beginning on the first postoperative day (POD) at 06:00 a.m. and continued during the following days until a normalization of the values was achieved. According to previous studies, we recorded the first postoperative value and the peak value of high*-*sensitivity cardiac troponin T (hs-cTnT) as well as creatine kinase (CK) and CK-MB as indicators for myocardial damage [13, 14]. As a safety endpoint, major adverse cardiac and cerebrovascular events (MACCE) were assessed, defined as a composite of myocardial infarction, stroke, or all-cause mortality. Moreover, we recorded the duration of ICU stay, in-hospital mortality, postoperative atrial fibrillation, and intraoperative parameters such as aortic cross-clamping time and number of distal anastomoses. Perioperative acute myocardial infarction was assessed by the treating clinicians (mainly on the intensive care unit) according to the recent guidelines [15–17].

**Figure 1:**
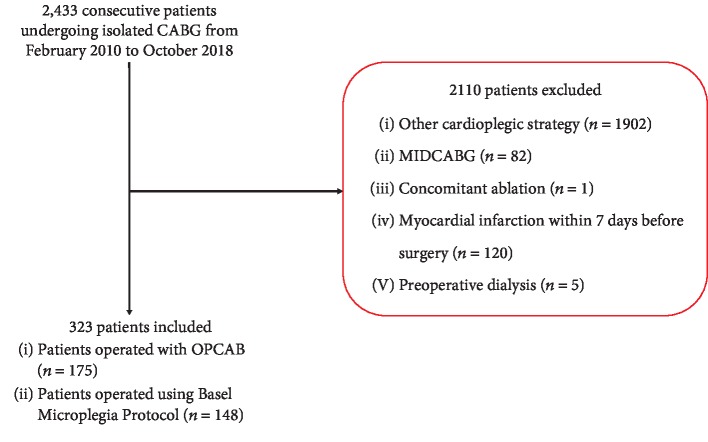
Patient flow chart.

Patients with nonstandard cardioplegic strategy, concomitant ablation, or previous myocardial infarction within 7 days before the operation, as well as patients undergoing minimally invasive direct coronary artery bypass grafting (MIDCAB), were excluded from this analysis (Figure 1).

### 2.3. Technical Aspects and Surgical Technique

Our techniques for OPCAB and isolated CABG using our novel microplegia protocol were previously described in detail [11, 13, 14]. All operations were performed through median sternotomy. When using the MiECC, the ascending aorta and the right atrium were cannulated after full heparinisation. Cardioplegic arrest was induced using microplegia after cross-clamping, as previously described [13, 14]. In brief, the microplegia (composed of patient's blood with potassium (K), magnesium (Mg), and lidocaine, thus normovolemic) is applied under pressure and flow control via the aortic root. The targeted flow is approximately 300 mL/min, and for safety reasons, the pressure is limited to 250 mmHg (measured directly in the MPS® console). The microplegia protocol consists of a 4-minute induction time (with reduced dosage of K after 2 minutes) and repetitive administration of 2 minutes every 20 minutes. Before declamping, a hot shot is given for 1 minute [13, 14].

The internal mammary artery (IMA), the radial artery, or the great saphenous vein were used as graft material. The vast majority of OPCAB procedures was performed by two experienced off-pump surgeons.

### 2.4. Statistical Analysis

**Figure 2:**
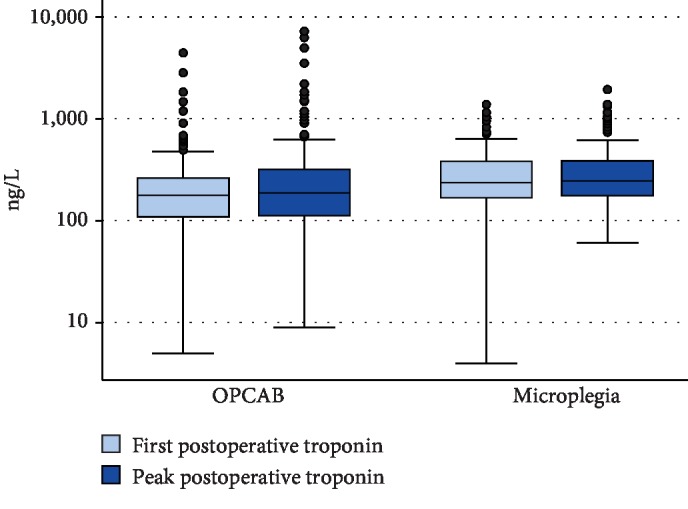
Boxplots hs-cTnT.

We conducted an inverse probability of treatment (IPTW) analysis and included age, body mass index, ejection fraction (EF), diabetes, three-vessel coronary artery disease, peripheral artery disease, preoperative stroke, preoperative renal failure, prior myocardial infarction (MI), hypertension, hypercholesterolemia, NYHA class 3 or 4, current smoking status, and EuroSCORE 2 as covariates into the propensity model. We trimmed the tails of the propensity score distribution according to Figure 1S (supplemental [Supplementary-material supplementary-material-1]). Differences between the treatment groups (microplegia and OPCAB) before and after IPTW were expressed as standardized differences to assess comparability independently of the number of observations; the standardized differences are displayed in Figure 2S (supplemental [Supplementary-material supplementary-material-1]). Standardized differences of ≤0.1 are considered sufficiently small to indicate no relevant differences. To investigate the impact of treatment on hs-cTnT, CK-MB, and CK, we used IPT-weighted median regression, given the skewed distribution of these cardiac markers. We included the number of distal anastomoses, duration of operation, main stem stenosis, and the use of both internal mammary arteries (BIMA) as covariates into median regression, as we expect the association between treatment and endpoint to be confounded by these intraoperative variables. We also show crude values before IPTW as median and interquartile range with a Kruskal-Wallis test for differences.

All other continuous data are reported as mean ± standard deviation comparisons being made using linear regression. However, length of stay on the ICU and hospital stay are shown as geometric means with confidence intervals that were back-transformed from the logarithmic scale (due to skewed distribution). Categorical data are reported as numbers with percentage and compared using logistic regression. Confidence intervals and *p* values are two-sided; a *p* value below 0.05 is considered significant. All analyses were performed by a biostatistician (BG) using Stata 15 (StataCorp, Texas).

## 3. Results

### 3.1. Preoperative Data (Table 1)

From February 2010 until October 2018, 2433 patients underwent isolated CABG surgery in our institution. 323 met the inclusion criteria and thus represent the cohort of this study (Figure 1). Before IPTW, patients undergoing OPCAB had significantly lower ejection fraction (EF), higher EuroSCORE 2, more peripheral artery disease, and more previous MIs. After IPTW, groups were comparable in regard to pretreatment characteristics.

After IPTW, 278 patients were analyzed, 153 of which had received OPCAB and 125 of which had received microplegia. Absolute standardized differences between treatment groups dropped below 0.1 for all baseline characteristics, indicating no relevant difference (Table 1).

### 3.2. Intraoperative Data (Table 2)

Before IPTW, patients undergoing CABG using microplegia were more likely to have three-vessel coronary artery disease and main stem stenosis, and they had longer operation times compared to patients undergoing OPCAB. Moreover, patients undergoing on-pump CABG had more distal anastomoses; the radial artery was more frequently used.

These differences maintained even after IPTW, indicating that they relate to the treatment itself rather than to patient characteristics. This supports the concept of including these variables as covariates. Intraoperative data are provided in Table 2.

### 3.3. Postoperative Data (Table 3)

No differences regarding in-hospital mortality, postoperative MI, postoperative stroke, or atrial fibrillation at discharge remained after IPTW. However, length of stay on the ICU was significantly shorter in patients operated using microplegia. Postoperative results are depicted in Table 3.

### 3.4. Endpoint Analysis (Table 4, Figures 2–4)

After IPTW, both median (IQR) POD1 and peak hs-cTnT values were numerically higher in the microplegia group than in the OPCAB group, but this difference did not reach statistical significance (POD1: 174 ng/L (73-274 ng/L) in the OPCAB group versus 213 ng/L (117-308 ng/L) in the microplegia group, *p* = 0.105; peak: 178 ng/L (68-289 ng/L) in the OPCAB group versus 213 ng/L (109-318 ng/L) in the microplegia group, *p* = 0.155). Median (IQR) CK-MB was significantly lower in patients undergoing OPCAB than in patients receiving microplegia (POD1: 6.7 *μ*g/L (1.3-12.1 *μ*g/L) versus 11.6 *μ*g/L (6.7-16.4 *μ*g/L); *p* < 0.001; peak: 8.0 *μ*g/L (2.7-13.4 *μ*g/L) versus 12.0 *μ*g/L (7.2-16.8 *μ*g/L), *p* = 0.002). Median (IQR) CK on POD1 was significantly higher in the microplegia group than in the OPCAB group (174 U/L (0-376 U/L) versus 295 U/L (113-478 U/L), *p* = 0.012), whereas median (IQR) peak CK values were higher in the OPCAB group than in the microplegia group; this difference, however, was not statistically significant (404 U/L (166-642 U/L) versus 371 U/L (156-585 U/L), *p* = 0.522).

## 4. Discussion

The aim of this study was to compare our institutionally refined microplegia, applied with the MPS® (Basel Microplegia Protocol), in patients undergoing isolated CABG surgery using the MiECC, with mere OPCAB revascularization. We report five major findings.

First, OPCAB as well as CABG using the Basel Microplegia Protocol is safe and feasible in isolated CABG surgery, with low in hospital mortality and low MACCE rates in both groups. Second, the number of distal anastomoses was significantly lower among patients undergoing OPCAB procedure than in patients operated on-pump. Third, cardiac markers were extraordinarily low in both groups. While there were no significant differences regarding POD1 and peak hs-cTnT, POD1 values of CK-MB and CK as well as peak values of CK-MB were significantly lower in the OPCAB group. Fourth, we found no significant differences regarding postoperative atrial fibrillation between the two groups. Fifth, length of stay on the ICU was significantly lower in patients undergoing MiECC assisted CABG surgery using the microplegia than in OPCAB patients.

These data corroborate our promising first results that we have achieved after having introduced our institutionally refined dose/volume-dependent microplegia applied with the MPS® in isolated CABG surgery using the MiECC [13, 14].

The question of whether to perform OPCAB or on-pump surgery has been the subject of controversy for decades. While OPCAB provides advantages due to the missing contact between blood and foreign material or air as well as due to the absence of aortic cannulation potentially causing embolisms, there are also concerns regarding the long-term patency rates and completeness of revascularization in OPCAB procedures [3, 8, 9, 18, 19]. Large randomized controlled trials failed to show a clear benefit for OPCAB procedures [3–6]. Moreover, after worse composite outcomes and poorer graft patency rates after one year in the Randomized On/Off Bypass (ROOBY) study, the ROOBY-FS study showed a higher prevalence of death from any cause and of the composite outcome consisting of death from any cause, repeat revascularization, and nonfatal myocardial infarction after 5 years in patients undergoing OPCAB [3, 7]. However, OPCAB may still be the preferred technique for experienced surgeons in selected patient cohorts, such as elderly, calcified, or high-risk patients [20–22].

Regarding our results, a few points merit consideration: First, the number of distal anastomoses was significantly lower in patients undergoing OPCAB, but still higher compared to other studies (Shroyer et al.: 2.9 ± 0.9; Parmeshwar et al.: 2.79 ± 0.8) [3, 23]. Lower numbers of anastomoses among patients undergoing OPCAB are a common finding; yet, it questions the completeness of revascularization [3]. However, there is also literature indicating that completeness of revascularization is dependent not only on the number of grafts performed but also on the number of grafts needed [24]. Second, we found no significant difference regarding postoperative hs-cTnT values between the groups, and the values were on a remarkably low level [13, 14]. Moreover, there were no differences regarding in-hospital mortality or MACCE. However, there is evidence for an association between high postoperative hs-cTnT values and adverse outcomes after on-pump cardiac surgery including CABG [25–27]. Therefore, we believe that optimizing cardioplegic solutions, reflected by low postoperative cardiac markers, is a crucial cornerstone in CABG surgery to provide the best possible outcomes. The low levels of cardiac markers are especially important when considering that OPCAB surgery in our clinic is only performed by two experienced off-pump surgeons, while on-pump CABG is an important part of the education of younger and unexperienced cardiac surgeons. The remarkably low values of cardiac markers achieved with our microplegia protocol allow for a good cardiac protection, even during longer operations with multiple anastomoses. Third, patients operated using microplegia were significantly shorter on the ICU than patients operated off-pump. This supports previous findings regarding shorter length of stay on the ICU after the use of microplegia [14, 28].

While hs-cTnT levels were similar in both groups, levels of CK-MB and CK on POD1 were significantly lower in the OPCAB group, indicating a possible increased myocardial damage in the microplegia group. Whether these differences are based on different pathophysiological mechanisms remains speculative.

Comparing these results to standard on-pump CABG surgery is difficult since various factors influence levels of cardiac markers. First, it was shown that the use of MiECC was associated with lower cTnI values compared to the use of ECC [29]. Moreover, also the use of the cardioplegia solution influences postoperative values of cardiac markers [14].

Some limitations warrant consideration when interpreting the findings of this study. First, it was an observational single-center study, which may compromise the external validity of our findings. Second, due to the propensity modelling method, the final study population is relatively small, which increases the risk of results being attributable to chance. Further studies with larger sample sizes will be more beneficial to enlighten the topic. On the other hand, the standardized differences after propensity modelling indicate that the treatment groups are very similar with respect to patient characteristics. Thus, differences observed during the postoperative course are likely related to the treatment, particularly because records that were suspicious for residual confounding have been trimmed. Third, the ingredients that were used as arrest agents (K, Mg, and lidocaine) have the drug approval and are licensed to be used in humans. A possible off-label use is to be considered [13].

## 5. Conclusion

The use of our institutionally refined microplegia in conjunction with the MiECC was associated with comparable results to OPCAB revascularization in regard to hs-cTnT while values of CK-MB and CK on POD1 were lower in the OPCAB group. MACCE were seen equally frequent in both patient groups, and patients could be discharged from the ICU earlier when microplegia was used than when OPCAB was applied.

## Figures and Tables

**Figure 3:**
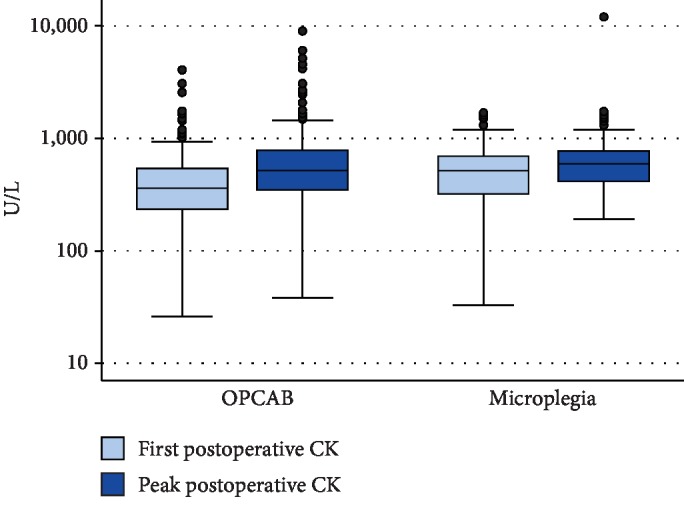
Boxplots CK.

**Figure 4:**
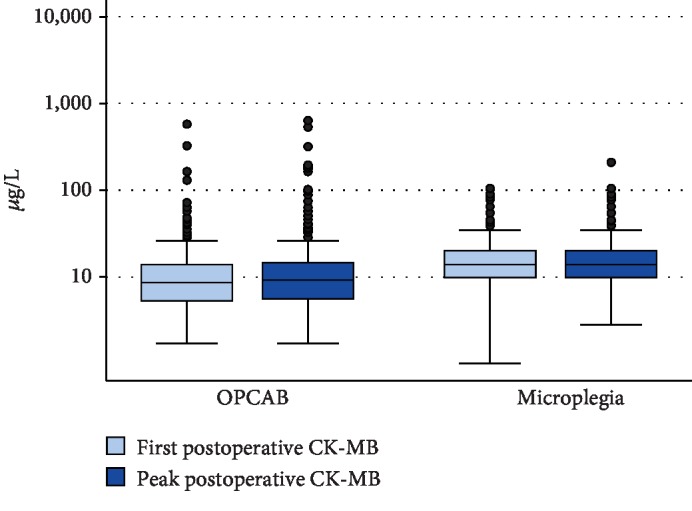
Boxplots CK-MB.

**Table 1:** Patient characteristics.

	Before IPTW				After IPTW			
	OPCAB (*n* = 175)	Microplegia (*n* = 148)	Diff.	*p*	OPCAB (*n* = 153)	Microplegia (*n* = 125)	Diff.	*p*
Age in years, m (SD)	69.3 (10.2)	68.0 (8.7)	-0.143	0.205	68.1 (10.4)	68.2 (9.7)	0.005	0.969
BMI in kg/m^2^, m (SD)	26.8 (4.0)	27.9 (4.7)	0.253	0.023	27.0 (4.3)	26.9 (5.1)	-0.017	0.890
Ejection fraction in %, m (SD)	53.0 (11.2)	56.3 (10.2)	0.306	0.007	54.4 (10.4)	54.2 (15.4)	-0.016	0.899
EuroSCORE 2, m (SD)	2.3 (2.0)	1.6 (1.6)	-0.383	0.001	1.9 (1.3)	1.8 (3.3)	-0.010	0.938
Female, *n* (%)	33 (18.9)	28 (18.9)	-0.002	0.989	27 (17.9)	23 (18.4)	-0.014	0.914
Diabetes, *n* (%)	57 (32.6)	60 (40.5)	-0.166	0.138	52 (33.9)	42 (33.5)	0.009	0.948
Peripheral artery disease, *n* (%)	45 (25.7)	22 (14.9)	0.272	0.018	32 (21.2)	25 (19.9)	0.033	0.811
Preoperative stroke, *n* (%)	24 (13.7)	11 (7.4)	0.205	0.074	17 (11.4)	11 (8.5)	0.096	0.460
Renal disease, *n* (%)	8 (4.6)	3 (2.0)	0.143	0.221	4 (2.4)	3 (2.3)	0.002	0.987
COPD, *n* (%)	18 (10.3)	17 (11.5)	-0.039	0.730	15 (9.7)	15 (12.1)	-0.077	0.546
Prior myocardial infarction, *n* (%)	75 (42.9)	44 (29.7)	0.276	0.015	60 (39.1)	49 (39.4)	-0.007	0.961
Hypertension, *n* (%)	158 (90.3)	126 (85.1)	0.157	0.160	135 (88.4)	110 (88.2)	0.006	0.964
Hypercholesterolemia, *n* (%)	141 (80.6)	113 (76.4)	0.103	0.357	120 (78.3)	100 (80.0)	-0.043	0.739
NYHA III or IV, *n* (%)	36 (20.6)	23 (15.5)	0.131	0.245	27 (17.9)	22 (17.7)	0.006	0.965
Preoperative atrial fibrillation, *n* (%)	9 (5.1)	9 (6.1)	-0.041	0.714	7 (4.5)	5 (4.4)	0.005	0.969
Current smoker, *n* (%)	31 (17.7)	29 (19.6)	-0.048	0.665	30 (19.8)	28 (22.1)	-0.056	0.689

Diff.: standardized differences. Data are presented as mean and standard deviation or as numbers (%). OPCAB: off-pump coronary artery bypass grafting; COPD: chronic obstructive pulmonary disease; IPTW: inverse probability of treatment weighting.

**Table 2:** Intraoperative data.

	Before IPTW				After IPTW		
	OPCAB (*n* = 175)	Microplegia (*n* = 148)	Diff.	*p*	OPCAB (*n* = 153)	Microplegia (*n* = 125)	Diff.	*p*
Number of distal anastomoses, m (SD)	3.2 (1.2)	3.8 (0.9)	0.613	0.000	3.3 (1.1)	3.7 (1.1)	0.370	0.002
Duration of operation in minutes, m (SD)	191.6 (42.9)	222.9 (50.2)	0.670	0.000	194.3 (42.7)	217.0 (63.4)	0.420	0.001
Three vessel coronary artery disease, *n* (%)	132 (75.4)	137 (92.6)	-0.481	0.000	132 (86.0)	105 (84.0)	0.057	0.696
Presence of main stem stenosis, *n* (%)	21 (12.0)	42 (28.4)	-0.417	0.000	19 (12.6)	37 (29.9)	-0.434	0.001
Total arterial revascularization, *n* (%)	45 (25.7)	34 (23.0)	0.064	0.568	34 (22.0)	30 (23.8)	-0.044	0.740
LIMA, *n* (%)	164 (93.7)	146 (98.6)	-0.260	0.041	145 (94.6)	123 (98.2)	-0.194	0.295
RIMA, *n* (%)	40 (22.9)	26 (17.6)	0.132	0.241	37 (24.4)	22 (17.8)	0.163	0.223
BIMA, *n* (%)	38 (21.7)	26 (17.6)	0.105	0.352	36 (23.4)	22 (17.8)	0.139	0.295
Use of the radial artery, *n* (%)	15 (8.6)	43 (29.1)	-0.543	0.000	15 (9.9)	33 (26.7)	-0.444	0.001
IV inotropes at end of operation, *n* (%)	32 (18.4)	27 (18.5)	-0.003	0.981	28 (18.1)	29 (23.0)	-0.121	0.383

Data are presented as mean and standard deviation or as numbers (%). Diff.: standardized differences; OPCAB: off-pump coronary artery bypass grafting; LIMA: left internal mammary artery; RIMA: right internal mammary artery; BIMA: both internal mammary arteries; IV: intravenous; IPTW: inverse probability of treatment weighting.

**Table 3:** Postoperative data.

	Before IPTW				After IPTW			
	OPCAB (*n* = 175)	Microplegia (*n* = 148)	Diff.	*p*	OPCAB (*n* = 153)	Microplegia (*n* = 125)	Diff.	*p*
Operative mortality, *n* (%)	6 (3.4)	0 (0.0)	0.266	0.033	4 (2.5)	0 (0.0)	0.228	0.130
MACCE, *n* (%)	14 (8.0)	6 (4.1)	0.166	0.150	12 (7.8)	6 (5.0)	0.113	0.507
Postoperative MI, *n* (%)	4 (2.3)	1 (0.7)	0.134	0.272	5 (3.0)	0 (0.0)	0.249	0.130
Postoperative stroke, *n* (%)	5 (2.9)	5 (3.4)	-0.030	0.788	5 (3.2)	6 (5.0)	-0.089	0.568
Pulmonary infection, *n* (%)	12 (6.9)	5 (3.4)	0.158	0.171	11 (6.9)	4 (3.3)	0.168	0.193
Postoperative renal failure, *n* (%)	15 (8.6)	5 (3.4)	0.220	0.062	13 (8.6)	3 (2.7)	0.255	0.077
AF at discharge, *n* (%)	34 (19.4)	35 (23.6)	-0.103	0.357	25 (16.5)	27 (21.9)	-0.136	0.281
Reoperation for bleeding, *n* (%)	3 (1.7)	5 (3.4)	-0.106	0.347	3 (1.7)	7 (5.8)	-0.218	0.157
Intubation > 72 h, *n* (%)	2 (1.1)	0 (0.0)	0.152	0.502	2 (1.1)	0 (0.0)	0.149	0.503
Length of ICU stay in days, geometric mean (CI)	1.7 (1.5-1.9)	1.3 (1.2-1.4)	0.423	0.003	1.6 (1.5-1.8)	1.3 (1.2-1.4)	0.419	0.014
Length of stay in days, geometric mean (CI)	8.5 (7.9-9.2)	8.7 (8.3-9.2)	0.658	0.399	8.7 (8.0-9.4)	8.8 (8.3-9.4)	0.646	0.395

Data are presented as geometric mean and standard deviation back-transformed from the log scale or as numbers (%). Diff.: standardized differences; OPCAB: off-pump coronary artery bypass grafting; MACCE: major adverse cardiac and cerebrovascular events; ICU: intensive care unit; MI: myocardial infarction; IPTW: inverse probability of treatment weighting.

**Table 4:** Cardiac markers.

	Crude nonparametric analysis, before IPTW			Adjusted, after IPTW			
	OPCAB (*n* = 175)	Microplegia (*n* = 148)	*p*	OPCAB (*n* = 153)	Microplegia (*n* = 125)	Effect on median	*p*
hs-cTnT, POD1 in ng/L, median (IQR)	176 (106-258)	225 (159-372)	<0.001	174 (73-274)	213 (117-308)	39 (-8-87)	0.105
Peak hs-cTnT in ng/L, median (IQR)	187 (112-318)	237 (167-380)	0.001	178 (68-289)	213 (109-318)	35 (-13-84)	0.155
CK-MB POD1 in *μ*g/L, median (IQR)	8.6 (5.3-13.9)	13.4 (9.3-19.7)	<0.001	6.7 (1.3-12.1)	11.6 (6.7-16.4)	4.9 (2.3-7.4)	0.000
Peak CK-MB in *μ*g/L, median (IQR)	9.4 (5.6-14.6)	13.4 (9.3-19.9)	<0.001	8.0 (2.7-13.4)	12.0 (7.2-16.8)	4.0 (1.4-6.5)	0.002
CK POD1 in U/L, median (IQR)	357 (233-542)	503 (322-691)	<0.001	174 (0-376)	295 (113-478)	121 (27-216)	0.012
Peak CK in U/L, median (IQR)	508 (339-783)	577 (401-768)	0.296	404 (166-642)	371 (156-585)	-34 (-137-70)	0.522

Note that average cardiac markers on the right hand side are estimated from median regression at the average of the covariates (main stem stenosis, use of BIMA, duration of operation, and number of distal anastomoses) after IPTW. Data are presented as median with an interquartile range. OPCAB: off-pump coronary artery bypass grafting; hs-cTnT: high*-*sensitivity cardiac troponin T; CK: creatine kinase; CK-MB: creatine kinase-myocardial type; POD: postoperative day; IPTW: inverse probability of treatment weighting.

## Data Availability

The data used to support the findings of this study have not been made available because of the local ethical regulations.

## Supplementary Materials

Supplementary Materials Figure 1S: distribution of the propensity score in the two treatment groups. The red lines indicate the cut-off points beyond which observations were dropped before analysis. Figure 2S: standardized differences.

## References

[1] Sousa-Uva M., Neumann F. J., Ahlsson A. (2019). 2018 ESC/EACTS guidelines on myocardial revascularization. *European Journal of Cardio-Thoracic Surgery*.

[2] Bakaeen F. G., Shroyer A. L. W., Gammie J. S. (2014). Trends in use of off-pump coronary artery bypass grafting: results from the Society of Thoracic Surgeons Adult Cardiac Surgery Database. *Journal of Thoracic and Cardiovascular Surgery*.

[3] Shroyer A. L., Grover F. L., Hattler B. (2009). On-pump versus off-pump coronary-artery bypass surgery. *The New England Journal of Medicine*.

[4] Lamy A., Devereaux P. J., Prabhakaran D. (2013). Effects of off-pump and on-pump coronary-artery bypass grafting at 1 year. *New England Journal of Medicine*.

[5] Diegeler A., Börgermann J., Kappert U. (2013). Off-pump versus on-pump coronary-artery bypass grafting in elderly patients. *The New England Journal of Medicine*.

[6] Lamy A., Devereaux P. J., Prabhakaran D. (2016). Five-year outcomes after off-pump or on-pump coronary-artery bypass grafting. *The New England Journal of Medicine*.

[7] Shroyer A. L., Hattler B., Wagner T. H. (2017). Five-year outcomes after on-pump and off-pump coronary-artery bypass. *The New England Journal of Medicine*.

[8] Lamy A., Devereaux P. J., Prabhakaran D. (2012). Off-pump or on-pump coronary-artery bypass grafting at 30 days. *The New England Journal of Medicine*.

[9] Puehler T., Haneya A., Philipp A. (2009). Minimal extracorporeal circulation: an alternative for on-pump and off-pump coronary revascularization. *The Annals of Thoracic Surgery*.

[10] van Boven W., Gerritsen W., Waanders F., Haas F., Aarts L. (2004). Mini extracorporeal circuit for coronary artery bypass grafting: initial clinical and biochemical results: a comparison with conventional and off-pump coronary artery bypass grafts concerning global oxidative stress and alveolar function. *Perfusion*.

[11] Reuthebuch O., Koechlin L., Gahl B. (2014). Off-pump compared to minimal extracorporeal circulation surgery in coronary artery bypass grafting. *Swiss Medical Weekly*.

[12] Winkler B., Heinisch P. P., Zuk G. (2017). Minimally invasive extracorporeal circulation: excellent outcome and life expectancy after coronary artery bypass grafting surgery. *Swiss Medical Weekly*.

[13] Koechlin L., Zenklusen U., Doebele T. (2018). Clinical implementation of a novel myocardial protection pathway in coronary artery bypass surgery with minimal extracorporeal circulation. *Perfusion*.

[14] Koechlin L., Rrahmani B., Gahl B. (2019). Microplegia versus Cardioplexol® in coronary artery bypass surgery with minimal extracorporeal circulation: comparison of two cardioplegia concepts. *The Thoracic and Cardiovascular Surgeon*.

[15] Thygesen K., Alpert J. S., White H. D., Joint ESC/ACCF/AHA/WHF Task Force for the Redefinition of Myocardial Infarction (2007). Universal definition of myocardial infarction. *Journal of the American College of Cardiology*.

[16] Thygesen K., Alpert J. S., Jaffe A. S. (2012). Third universal definition of myocardial infarction. *European Heart Journal*.

[17] Thygesen K., Alpert J. S., Jaffe A. S. (2018). Fourth universal definition of myocardial infarction (2018). *Journal of the American College of Cardiology*.

[18] Panday G. F. V., Fischer S., Bauer A. (2009). Minimal extracorporeal circulation and off-pump compared to conventional cardiopulmonary bypass in coronary surgery☆. *Interactive Cardiovascular and Thoracic Surgery*.

[19] Puehler T., Haneya A., Philipp A. (2011). Minimized extracorporeal circulation system in coronary artery bypass surgery: a 10-year single-center experience with 2243 patients. *European Journal of Cardio-Thoracic Surgery*.

[20] Puskas J. D., Thourani V. H., Kilgo P. (2009). Off-pump coronary artery bypass disproportionately benefits high-risk patients. *The Annals of Thoracic Surgery*.

[21] Tarakji K. G., Sabik JF 3rd, Bhudia S. K., Batizy L. H., Blackstone E. H. (2011). Temporal onset, risk factors, and outcomes associated with stroke after coronary artery bypass grafting. *Journal of the American Medical Association*.

[22] Blackstone E. H., Sabik J. F. (2017). Changing the discussion about on-pump versus off-pump CABG. *The New England Journal of Medicine*.

[23] Parmeshwar N., Fero K. E., Manecke G., Coletta J. M. (2019). Off-pump versus on-pump: long-term outcomes after coronary artery bypass in a veteran population. *Journal of Cardiothoracic and Vascular Anesthesia*.

[24] Magee M. J., Hebert E., Herbert M. A. (2009). Fewer grafts performed in off-pump bypass surgery: patient selection or incomplete revascularization?. *The Annals of Thoracic Surgery*.

[25] Gahl B., Göber V., Odutayo A. (2018). Prognostic value of early postoperative troponin T in patients undergoing coronary artery bypass grafting. *Journal of the American Heart Association*.

[26] Mauermann E., Bolliger D., Fassl J. (2017). Association of troponin trends and cardiac morbidity and mortality after on-pump cardiac surgery. *The Annals of Thoracic Surgery*.

[27] Mauermann E., Bolliger D., Fassl J. (2017). Postoperative high-sensitivity troponin and its association with 30-day and 12-month, all-cause mortality in patients undergoing on-pump cardiac surgery. *Anesthesia and Analgesia*.

[28] Gerdisch M. W., Robinson S., David G., Makepeace S., Ryan M. P., Gunnarsson C. (2018). Clinical and economic benefits of advanced microplegia delivery system in cardiac surgery: evidence from 250 hospitals. *Journal of Comparative Effectiveness Research*.

[29] Immer F. F., Ackermann A., Gygax E. (2007). Minimal extracorporeal circulation is a promising technique for coronary artery bypass grafting. *The Annals of Thoracic Surgery*.

