# Supplementary material for: Analysis of Myocardial Ischemia Parameters after Coronary Artery Bypass Grafting with Minimal Extracorporeal Circulation and a Novel Microplegia versus Off-Pump Coronary Artery Bypass Grafting
Source: Mediators Inflamm. 2020 Jan 25;2020:5141503. doi: 10.1155/2020/5141503 (PMC7056992; doi:10.1155/2020/5141503)
Supplement: Supplementary Materials — Figure 1S: distribution of the propensity score in the two treatment groups. The red lines indicate the cut-off points beyond which observations were dropped before analysis. Figure 2S: standardized differences. [file 5141503.f1.pdf]

Analysis of Myocardial Ischemia Parameters After Coronary Artery Bypass Grafting with Minimal Extracorporeal Circulation and a Novel Microplegia Versus Off-Pump Coronary Artery Bypass Grafting

Online Supplemental

Figure 1S. Distribution of the propensity score in the two treatment groups.

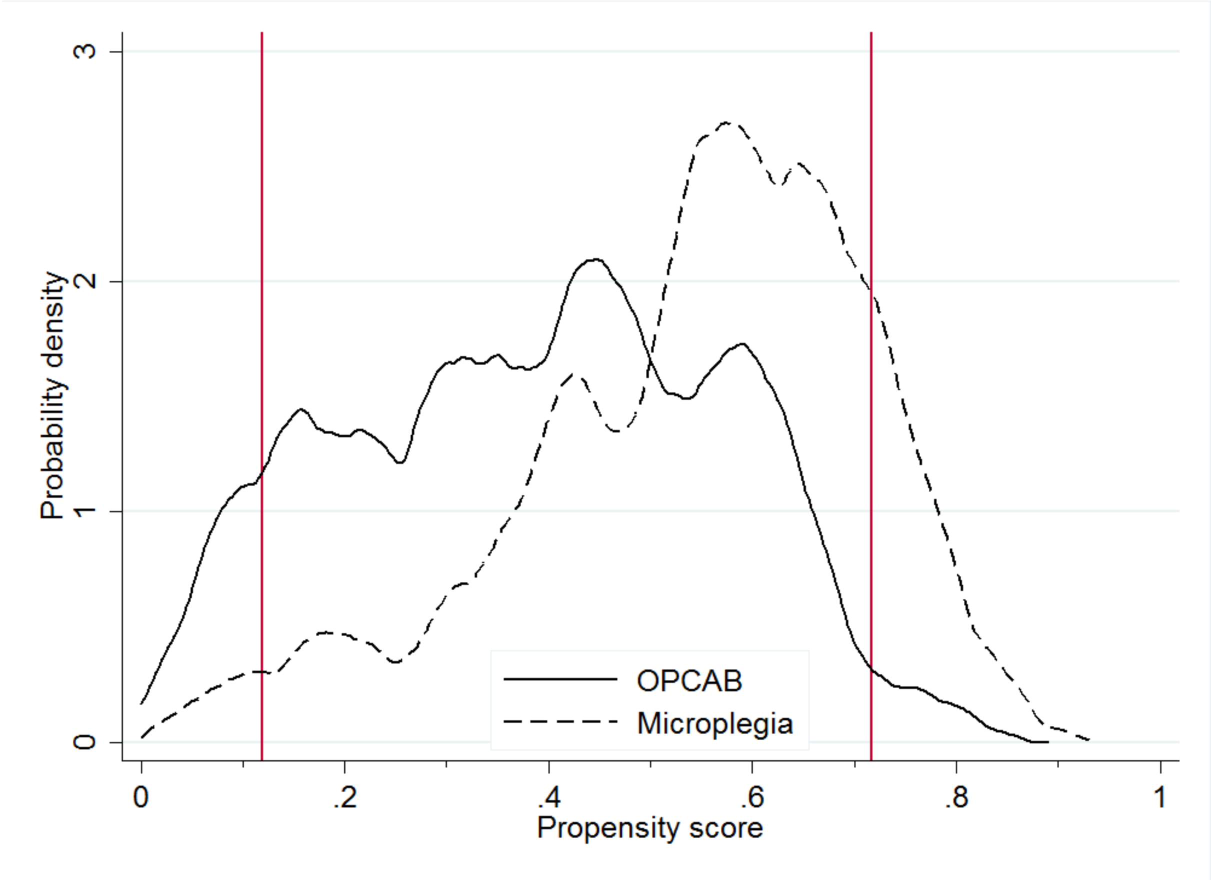

The red lines indicate the cut-off points beyond which observations were dropped before analysis.

Figure 2S. Standardized differences

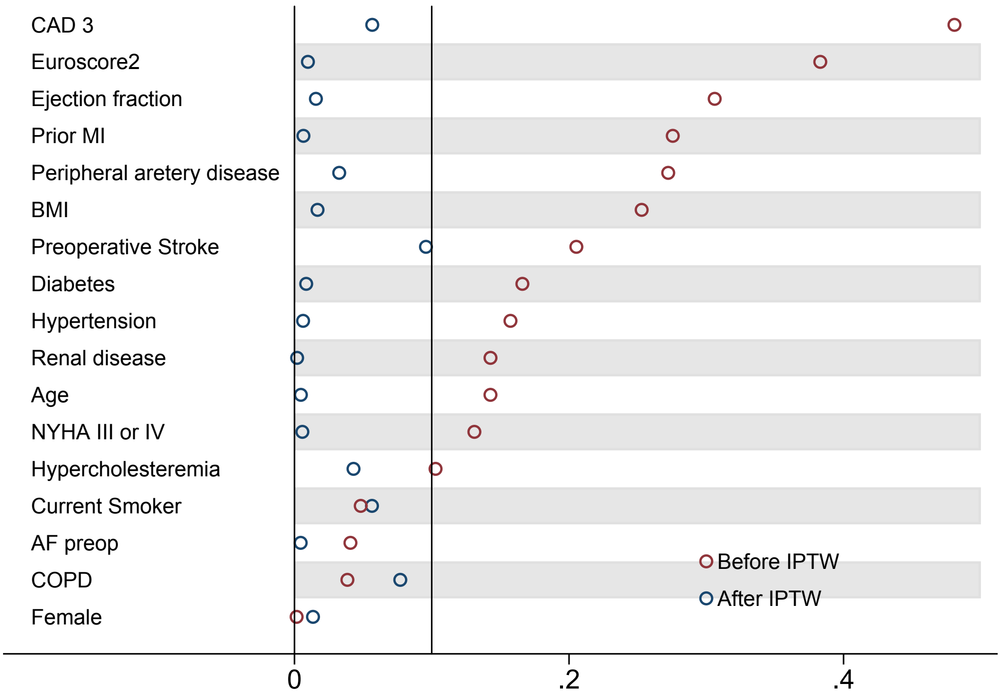

CAD:	Coronary artery disease
MI:	Myocardial infarction
BMI:	Body mass index
AF:	Atrial fibrillation
COPD:	Chronic obstrcutive pulmonary disease
